# Supplementary material for: Associations Between Uraemic Toxins and Gut Microbiota in Adults Initiating Peritoneal Dialysis
Source: Toxins (Basel). 2025 Jul 1;17(7):334. doi: 10.3390/toxins17070334 (PMC12300580; doi:10.3390/toxins17070334)
Supplement: Supplementary file 1 [file toxins-17-00334-s001.zip › toxins-3663606-supplementary.pdf]

SUPPLEMENTARY MATTER

Table S1: Study data obtained

Study data by type	N	Comments
Uraemic toxin data	43	ID 119 missing UT data
Patient reported symptoms	44	
Biochemistry (i.e. eGFR, albumin)	44	
Microbiota data	34	
Diet Data	27	
Complete diet and UT data	26	ID 119 missing UT data

UT: uraemic toxin

Table S2: Biochemical profile

	Minimum	Maximum	Median (IQR)	Mean (SD)
Electrolytes and Urea (mmol/l)				
Sodium	129	142	136 (134-139)	136.34 (3.45)
Potassium	3	6	4.2 (4-4.9)	4.36 (0.64)
Bicarbonate	16	30	23 (21-24)	22.82 (3.08)
Chloride	95	114	104 (100-107)	103.57 (4.68)
Urea	12	49	28 (24-32)	28.19 (8.25)
Kidney Function				
Creatinine µmol/l	342	1550	601 (514-795)	691.14 (268.46)
eGFR ml/min/1.73m^2	3	13	7 (5-9)	7.11 (2.54)
Protein				
Albumin (mmol/l)	16	39	31 (28-34)	30.91 (4.57)
Endocrine				
Corrected calcium mmol/l	1.88	3.17	2.36 (2.17-2.43)	2.33 (0.23)
Phosphate mmol/l	0.56	3.06	1.8 (1.6-2.1)	1.88 (0.58)
Parathyroid hormone ng/l	2.50	161.8	38.7 (23.1-58.1)	44.57 (31.32)
Haematology				
Haemoglobin (g/l)	66	143	101 (87-116)	101.47 (19.82)
White cell count (x10^9/l)	4.2	11.6	6.9 (6.1-8.4)	7.31 (1.94)
Platelet count (x10^9/l)	58	371	235 (180-287)	232.79 (72.88)
Iron Studies				
Ferritin (ng/ml)	21	2090	274 (125-450)	366.37 (390.84)
Transferrin (g/l)	1.4	45	22 (15-28)	21.41 (9.84)
Inflammatory Markers				
C-Reactive Protein (mg/l)	0	29	9 (4-12)	6 (7)

Table S3: Associations between uraemic toxins and macro- and micro-nutrients

	Total IS			Free IS			Total PCS			Free PCS			TMAO		
	Std β Coefficient	SE	P value	Std β Coefficient	SE	P value	Std β Coefficient	SE	P value	Std β Coefficient	SE	P value	Std β Coefficient	SE	P value
Vitamin B6 (mg/day)	-0.20	0.29	0.48	-0.17	0.28	0.54	-0.16	0.30	0.61	-0.13	0.29	0.65	-0.08	0.29	0.79
Calcium (mg/day)	0.05	0.28	0.86	0.10	0.27	0.71	0.04	0.29	0.89	0.07	0.28	0.80	0.12	0.28	0.68
Carbohydrate (g/day)	-0.08	0.58	0.90	0.08	0.56	0.88	-0.38	0.60	0.54	-0.19	0.58	0.74	-0.63	0.56	0.28
Fat (g/day)	0.18	0.53	0.74	-0.07	0.51	0.90	0.25	0.55	0.65	0.06	0.53	0.91	0.29	0.53	0.59
Dietary Fibre (g/day)	0.10	0.31	0.74	0.36	0.29	0.23	-0.54	0.30	0.09	-0.17	0.31	0.59	-0.08	0.31	0.81
Phosphorus (mg/day)	0.13	0.39	0.74	0.19	0.37	0.62	-0.07	0.40	0.87	0.00	0.39	1.00	0.27	0.38	0.49
Potassium (mg/day)	0.12	0.32	0.72	0.14	0.31	0.65	-0.21	0.33	0.53	-0.13	0.32	0.69	0.49	0.30	0.12
Protein-to-Fibre Ratio (g/g)	-0.09	0.27	0.75	-0.25	0.26	0.34	0.20	0.28	0.49	-0.02	0.27	0.95	0.14	0.27	0.60
Protein (g/day)	0.13	0.34	0.70	0.08	0.33	0.82	-0.16	0.35	0.66	-0.16	0.34	0.64	0.07	0.34	0.83
Saturated Fat (g/day)	0.37	0.32	0.25	0.42	0.30	0.18	0.30	0.33	0.37	0.35	0.32	0.29	0.09	0.33	0.78
Sodium (mg/day)	-0.25	0.32	0.45	-0.45	0.29	0.14	0.50	0.32	0.13	0.17	0.32	0.60	-0.09	0.32	0.78
Vitamin C (mg/day)	-0.06	0.23	0.82	0.10	0.23	0.67	-0.17	0.24	0.49	-0.02	0.24	0.95	0.24	0.23	0.30
Zinc (mg/day)	0.41	0.32	0.22	0.53	0.30	0.09	-0.16	0.35	0.65	0.05	0.34	0.88	0.56	0.31	0.09

Associations between uraemic toxins and daily nutrient intakes were assessed using linear regression.

β Coefficient represents the standardized beta coefficient (Std β), indicating the strength and direction of associations. Uraemic toxins were log-transformed, then both the independent and dependent variables were scaled and centred before model estimation. All associations were adjusted for age, gender, blood albumin levels, daily caloric intake, and diabetes diagnosis. Analyses were conducted using complete data from n=26 participants. Results are reported to two decimal places.
